# Remote patient monitoring and caregiver education for children with failure to thrive: a quasi-experimental study

**DOI:** 10.3389/fped.2026.1823172

**Published:** 2026-10-14

**Authors:** Jennifer Tisdale, Kaitlyn B. Newbaker, Joy B. Green, Regina QaDan, Md Rokibul Hasan, Lindsey B. Kuiper, Chris Collado, Vinayak K. Nahar, Jenna L. Dear

**Affiliations:** 1 Center for Children with Medical Complexity, Children’s of Mississippi, University of Mississippi Medical Center, Jackson, MS, United States; 2 School of Medicine, University of Mississippi Medical Center, Jackson, MS, United States; 3 Center for Telehealth, University of Mississippi Medical Center, Jackson, MS, United States; 4 Division of Pediatric Palliative Care, Children’s of Mississippi, University of Mississippi Medical Center, Jackson, MS, United States; 5 Division of Medicine, University of Mississippi Medical Center, Jackson, MS, United States; 6 Department of Dermatology, School of Medicine, University of Mississippi Medical Center, Jackson, MS, United States; 7 Department of Preventive Medicine, School of Medicine/John D. Bower School of Population Health, University of Mississippi Medical Center, Jackson, MS, United States

**Keywords:** children, failure to thrive, pediatrics, remote patient monitoring, telehealth

## Abstract

**Background:**

Failure to Thrive (FTT), also known as growth or weight faltering, is characterized by inadequate weight gain, poor growth, and potential developmental delays. Affecting up to 10% of outpatient pediatric populations and 5% of hospitalized children, FTT is particularly common among children with medical complexity. Traditional management depends on frequent in-person visits, which create barriers and delay interventions. To address these barriers, this study evaluated Remote Patient Monitoring (RPM) combined with caregiver education as a proactive, cost-conscious approach to FTT management.

**Methods:**

A quasi-experimental cohort study was conducted with children (ages 1–10 years) with FTT and requiring feeding tube support. Families transmitted weekly weight and feeding data using Bluetooth-enabled scales, supplemented by structured caregiver education. Outcomes included weight, hospital and emergency department (ED) utilization, and cost of care.

**Results:**

All 15 participants completed the six-month RPM program. Forty-two percent of participants (*n* = 6) achieved upward weight trends, 50% (*n* = 8) maintained stable weights, and 8% (*n* = 1) experienced a weight decline. Hospitalizations averaged 0.33 per patient pre-intervention, 0.60 during the intervention, and 0.33 post-intervention. Median hospitalizations were 0 (Q1 = 0, Q3 = 0) pre-intervention, 0 (Q1 = 0, Q3 = 1) during the intervention, and 0 (Q1 = 0, Q3 = 0.5) post-intervention. Emergency department visits averaged 1.07 pre-intervention, 0.60 during the intervention, and 0.87 post-intervention. Median ED visits were 0 (Q1 = 0, Q3 = 1) pre-intervention, 0 (Q1 = 0, Q3 = 1) during the intervention, and 1 (Q1 = 0, Q3 = 2) post-intervention. Median costs increased during the intervention phase before declining in the post-intervention period, despite mean costs being skewed upward by high utilizers.

**Conclusion:**

An RPM program integrated with caregiver education demonstrated feasibility in supporting proactive management of pediatric FTT. The intervention supported weight monitoring in this medically complex pediatric population, although healthcare utilization and costs varied across study periods. These preliminary findings support RPM may be a useful approach for managing pediatric FTT.

## Introduction

Failure to thrive (FTT), increasingly referred to as growth faltering, weight faltering, or poor weight gain, is a clinical condition characterized by inadequate weight gain, suboptimal growth, and/or developmental delays in infants and young children. The prevalence of FTT is estimated to be as high as 10% in outpatient pediatric populations and approximately 5% in hospitalized children (1, 2). Notably, children with special healthcare needs, chronic complex conditions, or medical complexities account for a significant proportion of these cases due to the heterogeneity of their feeding difficulties and the complexity of their caloric requirements (3, 4).

Importantly, FTT is not a discrete diagnosis but rather a clinical manifestation of underlying medical, nutritional, or developmental conditions. If left unaddressed, FTT can result in profound and long-term consequences, including delayed developmental milestones, impaired immune function, and heightened susceptibility to infections (2). Consequently, timely identification of the etiologies contributing to FTT, coupled with early and tailored interventions, is critical. Healthcare providers play a pivotal role in diagnosing the root causes of FTT and delivering comprehensive care that encompasses nutritional, medical, and developmental support to mitigate its impact (2).

Children with medical complexity (CMC) are a uniquely vulnerable subset of the pediatric population, as defined by the American Academy of Pediatrics (5). These children present with significant chronic health problems, functional limitations, and high healthcare and resource utilization (6). Despite comprising only 6% of Medicaid-insured pediatric patients, CMC accounts for nearly 40% of pediatric healthcare costs, 41% of pediatric hospital days, and 40% of pediatric hospital deaths (Figure 1) (7, 8). Among this population, FTT remains a prevalent and pressing challenge, further compounding the complexity of their care.

**Figure 1:**
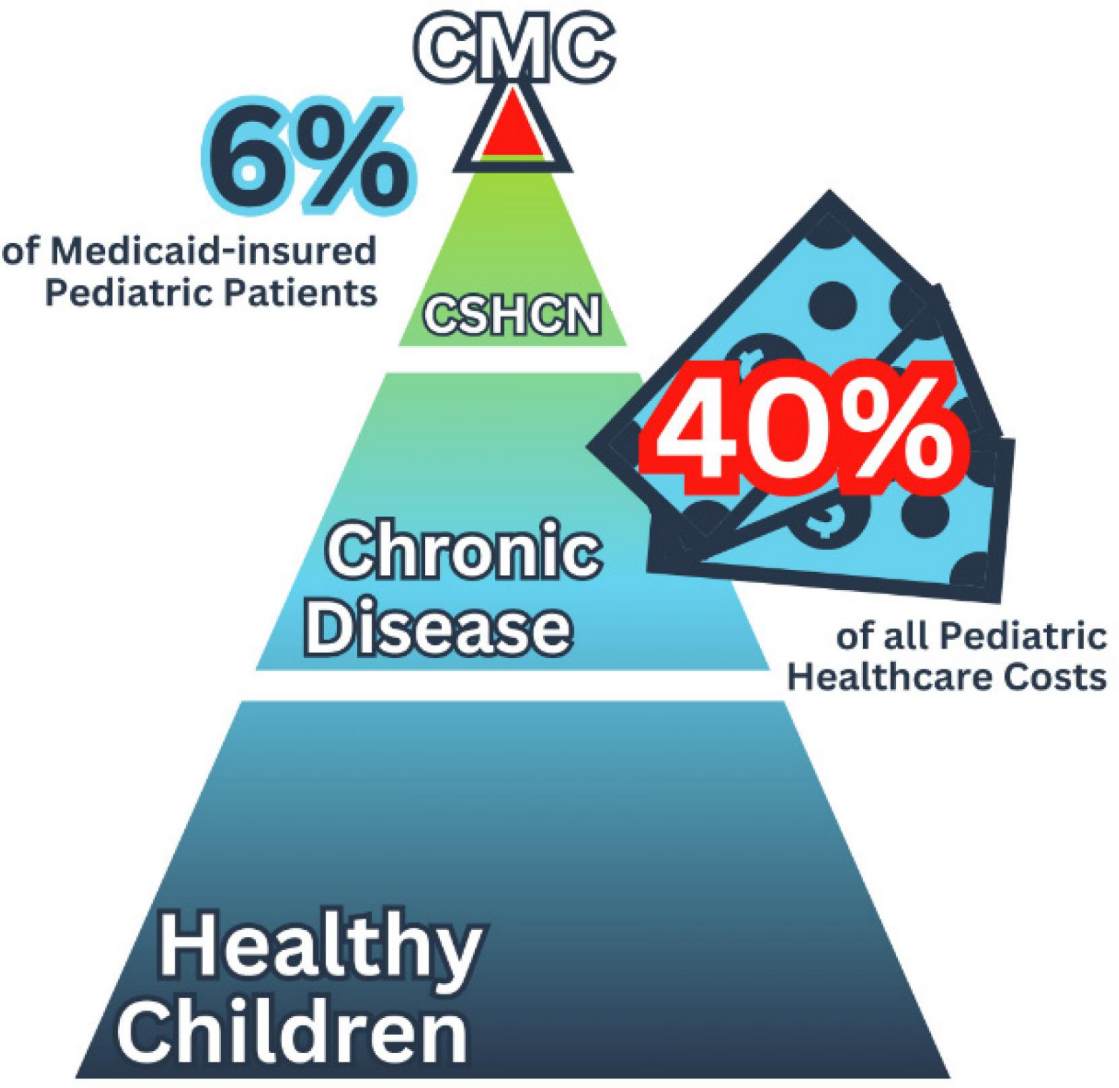
Distribution and healthcare burden of children with medical complexity. CMC, children with medical complexity; CSHCN, children with special health care needs.

Traditional management strategies often rely on frequent in-person visits that impose significant logistical and socioeconomic burdens on families. These barriers may lead to poor compliance, delayed interventions, and suboptimal outcomes. Limited research exists on how technology-enabled solutions such as Remote Patient Monitoring (RPM) could address these gaps for pediatric FTT, particularly in children with medical complexity.

This study was designed to evaluate whether RPM, integrated with structured caregiver education, could support weight outcomes and healthcare utilization in children with medical complexity. By enabling real-time monitoring, early identification of weight loss trends, and timely intervention, RPM has the potential to strengthen caregiver engagementand enhance continuity of care.

At Children's of Mississippi, the Complex Care team in the Division of Pediatric Palliative Care is a multidisciplinary care team that manages more than 1,400 children with medical complexities, with approximately 250 actively followed in the outpatient setting for FTT. Each month, an additional 4–5 children are admitted with FTT-related symptoms, and nearly half of the total patient population is identified as being at risk for developing FTT. Recognizing the urgent need for early intervention and more effective care strategies, the Complex Care team implemented a novel RPM program to address the unique challenges associated with FTT in this high-risk population. The program leveraged Bluetooth-enabled technology to facilitate follow-up visits, a critical component of weight monitoring and nutritional assessment that often imposes significant burdens on caregivers and contributes to poor compliance and suboptimal outcomes (9).

Through the RPM program, families monitored and transmitted key health metrics, such as weight and feeding patterns, directly to the care team in real time. This process enabled clinicians to provide timely, targeted interventions and maintain continuity of care between in-person visits. Caregivers also received comprehensive education to support their understanding of the technology and reinforce best practices for managing their child's condition.

The primary objective of the program was to evaluate whether this innovative approach could improve health outcomes compared to traditional in-person care. The study also aimed to identify factors contributing to FTT in children with medical complexity and to enhance understanding of the prevalence, causes, and barriers to treatment. In doing so, the initiative created a framework for integrating RPM and structured caregiver education into the management of pediatric FTT, particularly among children with medical complexity.

## Methods

### Study design

This study employed a quasi-experimental, pre- and post-comparison design with a quantitative focus to evaluate RPM in children with FTT. The intervention was implemented in a home-based setting to collect weight and feeding behavior data remotely, with the primary outcomes including weight stabilization, growth trajectory improvement, and reduction in acute care utilization, including hospitalizations and emergency department visits.

### Participants and eligibility

Participants were recruited through the Complex Care outpatient program at Children's of Mississippi between January 24, 2024, and November 4, 2024. Recruitment was based on clinic assessments, during which Complex Care providers offered families the option to participate. Written informed consent was obtained at the time of enrollment. Each participant was followed for six months from the signed consent date. Eligibility criteria included children aged 1–10 years, with a diagnosis of FTT, and reliance on feeding tube support. Caregivers were required to have access to a smartphone or cellular service; for those without access, a tablet device with cellular connectivity was provided. Exclusion criteria included children younger than 1 or older than 10 years, patients not requiring feeding tube support, and caregivers residing in areas without reliable cellular connectivity despite the provision of a study tablet.

### Intervention and data collection

Once enrolled, families completed baseline assessments that captured demographic information, medical history, and growth metrics (weight, height, and feeding behaviors). Caregivers received training on RPM devices, including Bluetooth-enabled scales connected to secure mobile applications for data submission (Figure 2). The Complex Care team—a multidisciplinary group of physicians, nurses, and care coordinators specializing in the management of medically complex children—monitored submissions remotely. RPM data were automatically transmitted directly into the electronic medical record (EMR) each night, where they were available for review by the Complex Care team. Interventions were delivered virtually or during scheduled ambulatory visits as needed.

**Figure 2:**
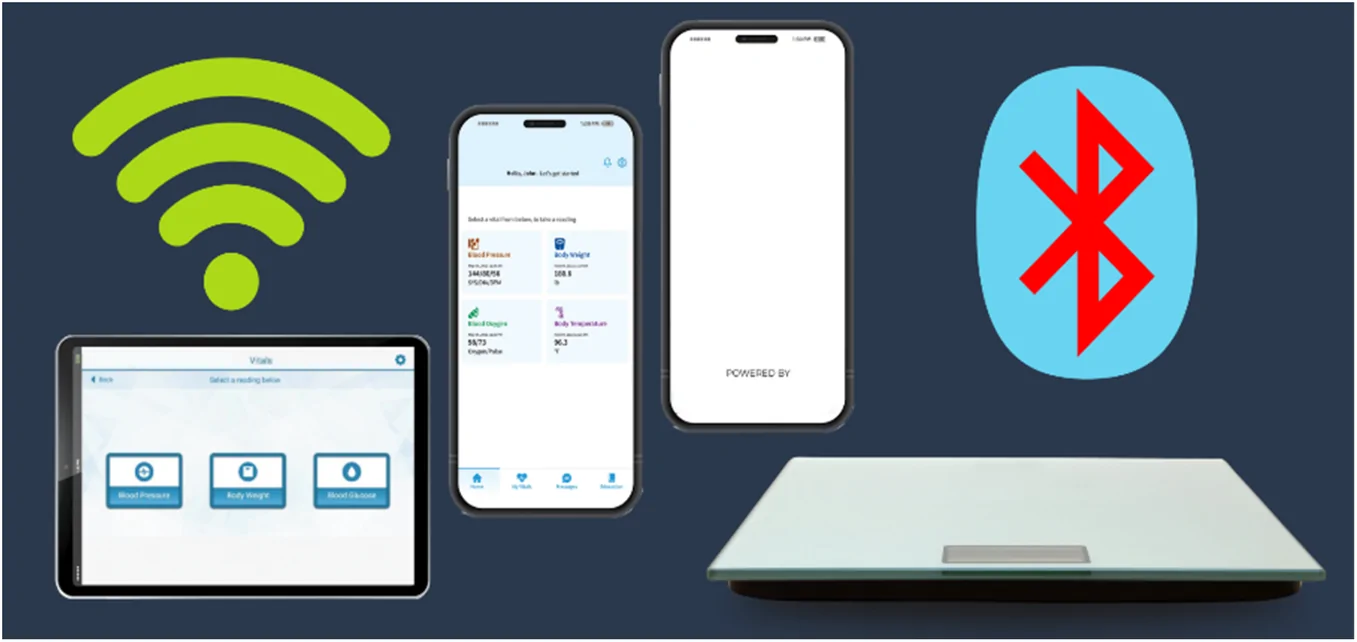
Bluetooth-enabled scale connects to mobile enabling caregivers to track and submit body weight. Caregivers had the option to BYOD (Bring Your Own Device) if they had a reliable smartphone and cellular service. For those without these resources, a tablet with cellular access was provided to ensure participation.

Caregivers were engaged through weekly RPM sessions that required submission of weight and feeding data. These sessions were supplemented with structured caregiver education, which provided individualized nutritional guidance, addressed barriers to participation, and reinforced engagement. Weekly educational support was designed to improve adherence, reduce preventable setbacks, and strengthen caregiver confidence in managing complex feeding regimens.

### Statistical analysis

Categorical variables (e.g., gender, race, caregiver education, household income) were described using frequencies and percentages. Continuous variables were summarized using mean (SD) and median (Q1, Q3), while categorical variables were described with counts (percentages). Patient outcomes are reported across three 6-month periods: pre-intervention, during the program, and post-intervention.

### Ethical approval

This study was reviewed and approved by the University of Mississippi Medical Center Institutional Review Board (IRB), and all procedures complied with ethical standards for human subjects research (IRB-Protocol-2023-243).

## Results

### Participant characteristics

All 15 participants completed the six-month RPM program. Table 1 shows socio-demographic characteristics of the participants. Weight outcomes demonstrated that 42% (*n* = 6) of participants achieved upward weight trends and 50% (*n* = 8) maintained stable weights, indicating nutritional stabilization through the intervention. One participant (8%) experienced weight decline associated with a vomiting illness and lower adherence to monitoring (Figure 3).

**Table 1:** Socio-demographic characteristics of the participants and caregiver households (*n* = 15).

Gender	n (%)
Male	5 (33.3)
Female	10 (66.7)
Income^a^	
$10,000–$24,999	11 (73.3)
$75,000–$99,999	1 (6.7)
$150,000+	1 (6.7)
Prefer not to answer	2 (13.3)
Race/Ethnicity	
White	6 (40.0)
Black/African American	8 (53.3)
American Indian/Alaskan Native	1 (6.7)
Education	
High School Diploma or equivalent	6 (40.0)
Some college	6 (40.0)
Less than High School	2 (13.3)
Bachelor's Degree or higher	1 (6.7)

a Household income and education represent caregiver-reported characteristics.

**Figure 3:**
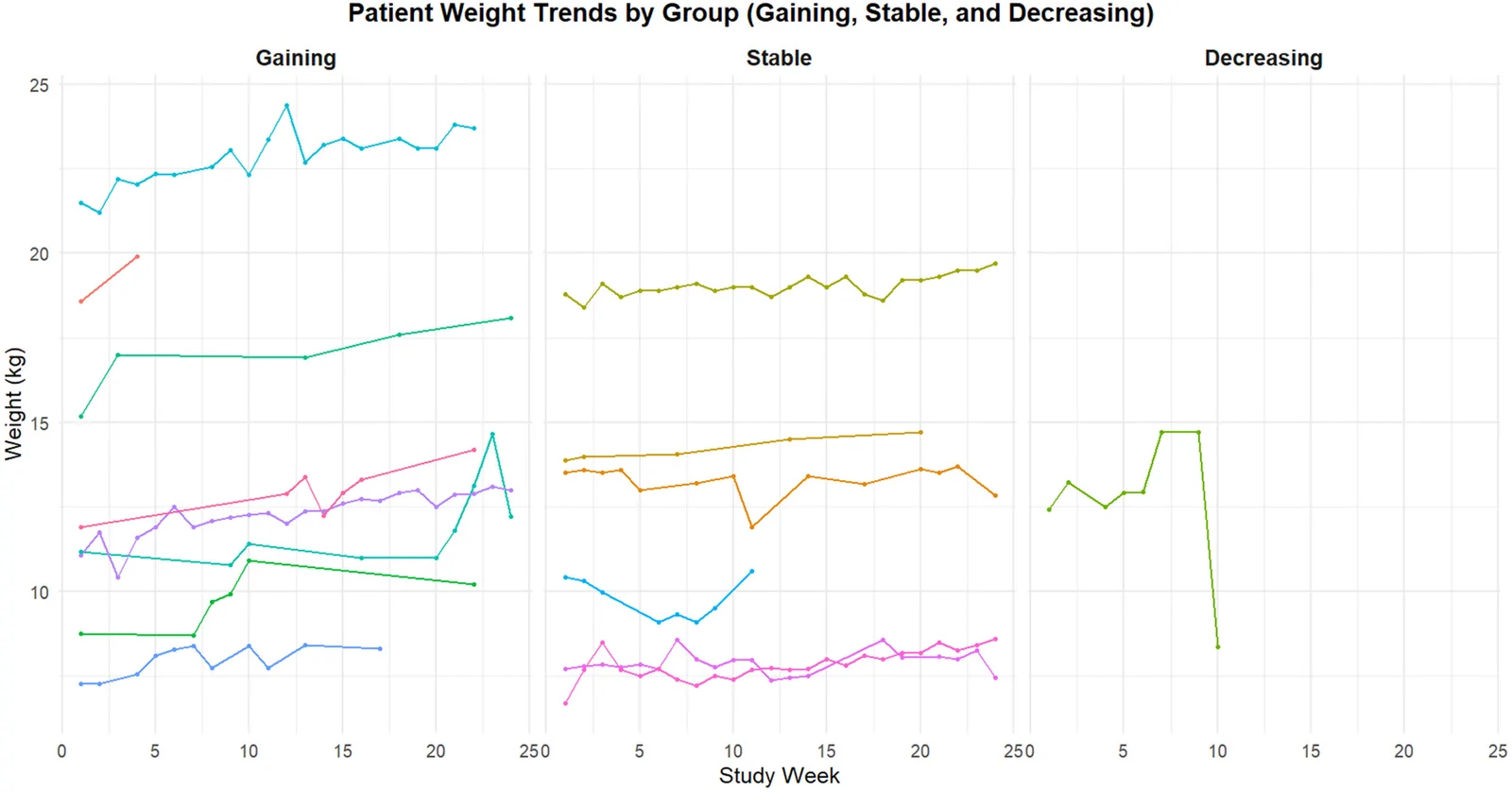
Patient weight trends during the remote patient monitoring program, categorized by gaining, stable, and decreasing groups.

### Hospital and emergency department utilization

Hospital and ED utilization are summarized in Table 2. The mean number of hospitalizations per patient was 0.33 (SD 0.72) pre-intervention, 0.60 (SD 0.83) during the intervention, and 0.33 (SD 0.62) post-intervention. Median hospitalizations were 0 (Q1 = 0, Q3 = 0) pre-intervention, 0 (Q1 = 0, Q3 = 1) during the intervention, and 0 (Q1 = 0, Q3 = 0.5) post-intervention. Emergency department visits varied across study periods, with mean values of 1.07 (SD 2.12) pre-intervention, 0.60 (SD 1.06) during the program, and 0.87 (SD 0.92) post-intervention. Median ED visits were 0 (Q1 = 0, Q3 = 1) pre-intervention, 0 (Q1 = 0, Q3 = 1) during, and 1 (Q1 = 0, Q3 = 2) post-intervention. These results indicate that while most participants had few or no hospitalizations or ED visits, a small subset of higher utilizers contributed disproportionately to overall totals.

**Table 2:** Changes in hospitalizations, emergency department visits, and cost per patient across pre-, during-, and post-intervention phases.

Outcome	Period	Mean (SD)	Median (Q1, Q3)	Total
Hospitalizations	Pre (6 mo)	0.33 (0.72)	0 (0, 0)	5
	During (6 mo)	0.60 (0.83)	0 (0, 1)	9
	Post (6 mo)	0.33 (0.62)	0 (0, 0.5)	5
Emergency Department Visits	Pre (6 mo)	1.07 (2.12)	0 (0, 1)	16
	During (6 mo)	0.60 (1.06)	0 (0, 1)	9
	Post (6 mo)	0.87 (0.92)	1 (0, 2)	13
Cost per patient (USD)^a^	Pre (6 mo)	$5,191 (6,271)	$2,840 (677, 7,801)	$77,871
	During (6 mo)	$8,047 (11,358)	$3,329 (462, 10,287)	$120,710

a Costs represent insurer-paid amounts (allowed claims) derived from Epic claims data. Values reflect standardized 6-month pre- and during-intervention periods for all 15 participants.

### Healthcare costs

Cost of care was calculated using insurer-paid amounts derived from Epic claims data, reflecting actual reimbursements to the hospital. The mean per-patient cost was $4,630 (SD $5,346) pre-intervention, increased to $7,346 (SD $11,000) during the intervention, and then decreased to $6,405 (SD $13,298) in the post-intervention period. Median costs changed from $2,308 (Q1=$677, Q3=$6,949) pre-intervention to $3,268 (Q1=$276, Q3=$8,601) during intervention, and reduced to $1,462 (Q1=$560, Q3=$3,476) post-intervention—demonstrating that while a few patients generated higher expenditures, most families experienced substantially lower costs after the end of the program.

### Influence of high utilizers

Notably, costs were disproportionately influenced by a small number of high utilizers. During the intervention, the highest-costing patient ($36,717) accounted for 3 of the 13 hospitalizations and 3 of the 10 ED visits. The same participant who had the highest healthcare costs during the intervention period also accounted for the single ED-to-hospital admission responsible for 51% of post-intervention period costs. These findings underscore that while mean costs were elevated by outliers, median costs more accurately reflect the typical patient experience, which showed reductions in both expenditures and acute care encounters.

## Discussion

The study results underscore several important implications for optimizing the use of RPM in pediatric care, particularly for children with Failure to Thrive (FTT). By facilitating early and frequent monitoring between periodic clinic visits, the RPM program shifted the model of care from reactive, hospital-based interventions toward proactive engagement. This approach not only supported timely adjustments to care plans but also provided families with tools to manage their child's condition more effectively, reducing reliance on emergency interventions.

Notably, the cost of care demonstrated substantial variation across the three study periods. When examining total insurer-paid costs across the full study timeline—including early hospitalizations and the monthly cost of RPM services—pre-intervention costs totaled $77,871, rising to $120,710 during the intervention phase before dropping sharply to $20,489 in the post-intervention period. Cost estimates were influenced by a small number of high-utilizing participants and should therefore be interpreted cautiously. Healthcare utilization also varied across the three study periods. Hospitalizations increased from 5 pre-intervention to 9 during the intervention and returned to 5 post-intervention. Emergency department visits decreased from 16 pre-intervention to 9 during the intervention before increasing to 13 post-intervention. These findings demonstrate variability in healthcare utilization and costs across study periods and should be interpreted cautiously given the small sample size and absence of a control group. Clinical outcomes further reinforce the utility of this approach: 42% of participants achieved weight gain and 50% maintained stable weights, suggesting that RPM contributed to improved nutritional trajectories. However, adherence challenges impacted data completeness. While the monitoring system consistently captured reliable growth data when entries were submitted, gaps arose due to caregiver noncompliance with scheduled weigh-ins and educational surveys—barriers that were likely influenced by socioeconomic and technological factors.

Qualitative insights provide additional context. Although end-of-study surveys were not completed by most caregivers, limiting systematic assessment of satisfaction, individual caregiver testimonials were informative. One caregiver described two-way communication with the care team as particularly valuable for addressing non-emergent questions and noted that timely adjustments were made when their child experienced weight decline due to a vomiting illness. Such feedback highlights the potential of RPM to strengthen family–provider communication even in the absence of in-person visits, while also underscoring the importance of integrating strategies to encourage survey participation in future studies.

### Compliance and completeness

- Growth Data Completeness: All complete data points reflected the program’s robust monitoring capacity; however, missing entries were primarily due to caregiver noncompliance with scheduled weigh-ins.
- Adherence Challenges: Inconsistent participation in weekly weigh-ins and educational surveys was linked to socioeconomic barriers and technological difficulties, emphasizing the need for simplified tools and enhanced caregiver engagement strategies.

### Limitations and future directions

Several limitations must be acknowledged. This was a small pilot study with only 15 participants, which limits the generalizability of the findings. Because of the exploratory nature of this pilot study and the small sample size, the analyses were descriptive and not intended to establish statistically significant differences. Moreover, recruitment through provider referral may also have introduced selection bias, and data on eligible families who declined participation were not systematically collected. The presence of outliers, including a single participant who accounted for a substantial proportion of post-intervention healthcare costs, may have disproportionately influenced cost estimates. Accordingly, median cost estimates should be interpreted alongside mean values, as medians are more robust to extreme observations and may better represent the typical patient experience in this small cohort. In addition, the broad age range of participants (1–10 years) may have influenced the results because children at different ages have different medical needs and growth patterns. Therefore, the findings should be considered preliminary and interpreted with caution until confirmed by larger studies. Participants demonstrating stable or improved growth were more likely to adhere to weekly weigh-ins and educational sessions, while socioeconomic challenges and technological barriers hindered adherence in others. These findings underscore the need for tailored support to enhance participation.

The RPM program nonetheless demonstrated significant promise. By enabling early identification of weight loss trends, reducing readmissions, and supporting growth outcomes, it offers a scalable model for managing FTT in children with medical complexity. These findings are consistent with previous reports demonstrating the feasibility and value of pediatric remote patient monitoring (RPM), including improved caregiver communication, enhanced access to care, and timely clinical intervention through remote monitoring (10–12). Future work should focus on simplified technology interfaces, expanded caregiver education, and earlier initiation of RPM—for example, at the time of hospital discharge for newly diagnosed children with FTT —to strengthen caregiver engagement and ensure consistent monitoring during critical recovery phases. Expanding the cohort to a larger and more diverse population will be essential to evaluate scalability, long-term financial sustainability, and cost-effectiveness using more robust statistical methods.

By addressing adherence barriers and refining program elements, RPM has the potential to become a sustainable, technology-supported framework for pediatric FTT management. This approach can help meet the multifaceted needs of children with medical complexity by combining proactive monitoring, timely intervention, and enhanced caregiver engagement.

## Data Availability

The raw data supporting the conclusions of this article will be made available by the authors, without undue reservation.
